# Supplementary material for: Differentiation of Baboon (Papio anubis) Induced-Pluripotent Stem Cells into Enucleated Red Blood Cells
Source: Cells. 2019 Oct 19;8(10):1282. doi: 10.3390/cells8101282 (PMC6829891; doi:10.3390/cells8101282)
Supplement: Supplementary file 1 [file cells-08-01282-s001.pdf]

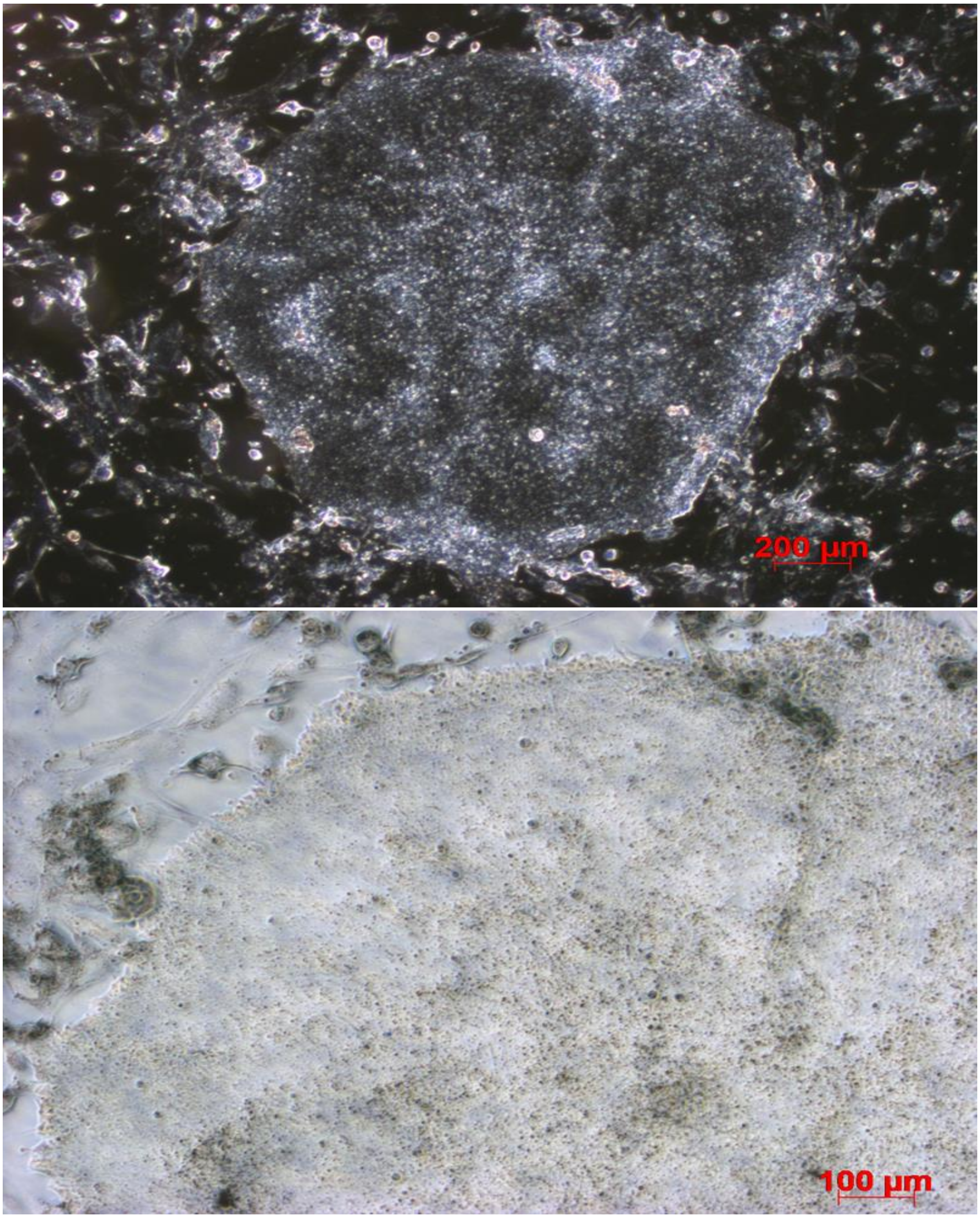

Figure S1a: Morphology of baboon iPSCs grown on a MEF feeder layer : 2.5x(top) and 5x (bottom) magnification phase contrast micrograph illustrating the typical morphology of baboon iPSCs.

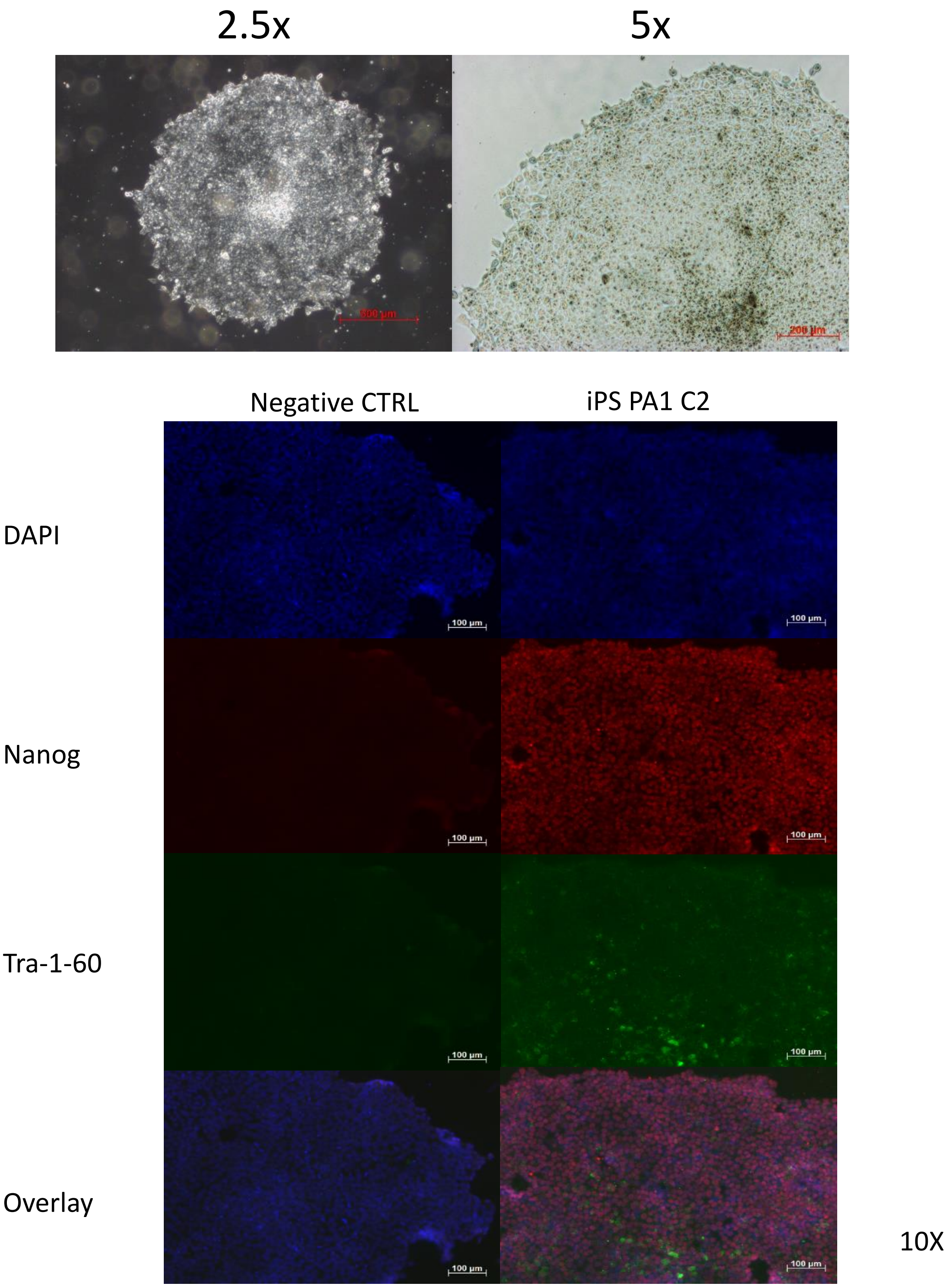

Figure S1b: **Morphology of baboon iPSCs grown on E8/vitronectin** Top: Phase contrast micrographs of baboon iPSCs grown on vitronectin .Bottom: immuno staining of baboon iPSCs grown on vitronectin and stained with DAPI, Nanog (Alexa-fluor 555) and Tra-1-60 (Alexa-fluor 488).
All cells express nanog but only a subset express Tra-1-60.

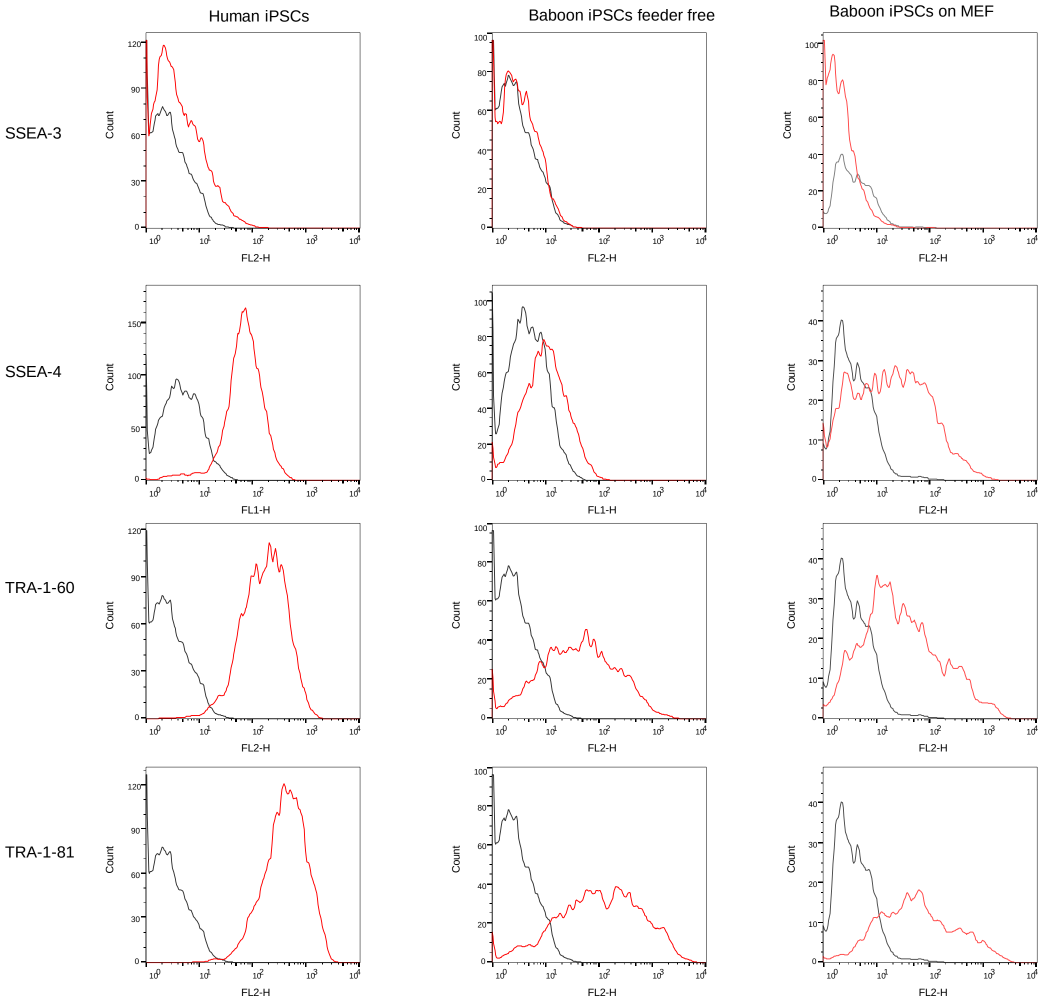

Figure S2:FACS analysis of human and baboon iPSCs grown in chemically-defined-conditions. Black histogram: isotype controls; red histograms: baboon or human iPSCs. Baboon iPSCs express pluripotency markers albeit at lower levels than human iPSCs.

a

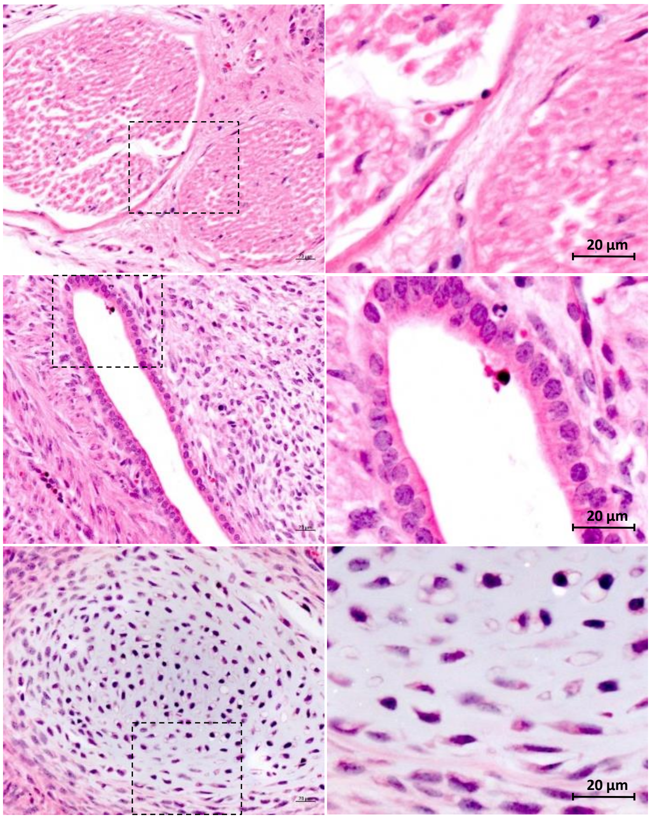

Ectoderm – peripheral nerve

Endoderm – glandular epithelium

Mesoderm - cartilage

Figure S3a-c: Teratoma analysis. 1x10^{6} baboon iPSCs were injected intramuscularly into the hind leg of a 6–8 week old NSG mouse. Six week later, tumors were fixed in 10% formalin, paraffin embedded, sectioned and stained with hematoxylin/eosin. Results from multiple tumors obtained from multiple iPSCs are shown. Structures originating from all three germ layers were found in most tumors analyzed

b

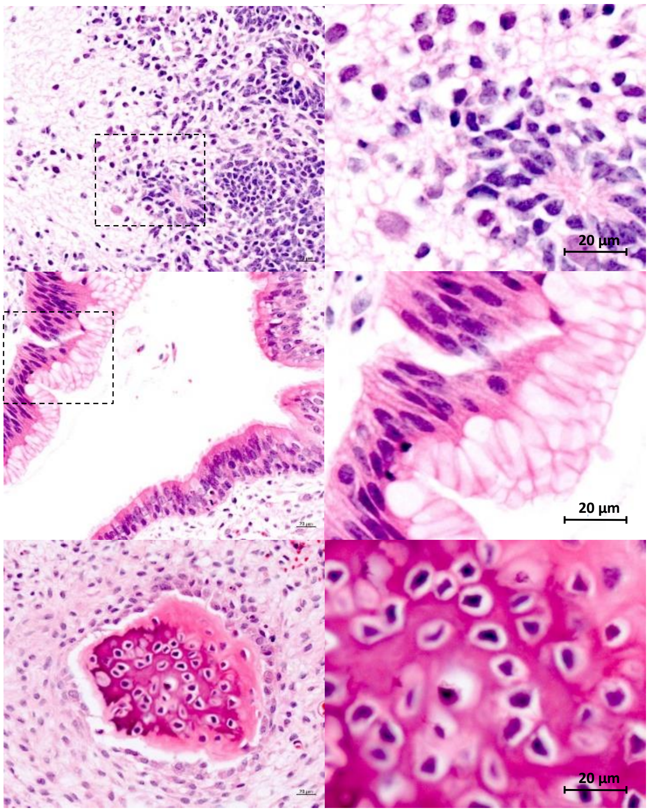

Ectoderm – nervous tissue

Endoderm - respiratory epithelium

Mesoderm – bone from a cartilage model

Figure S3b

c

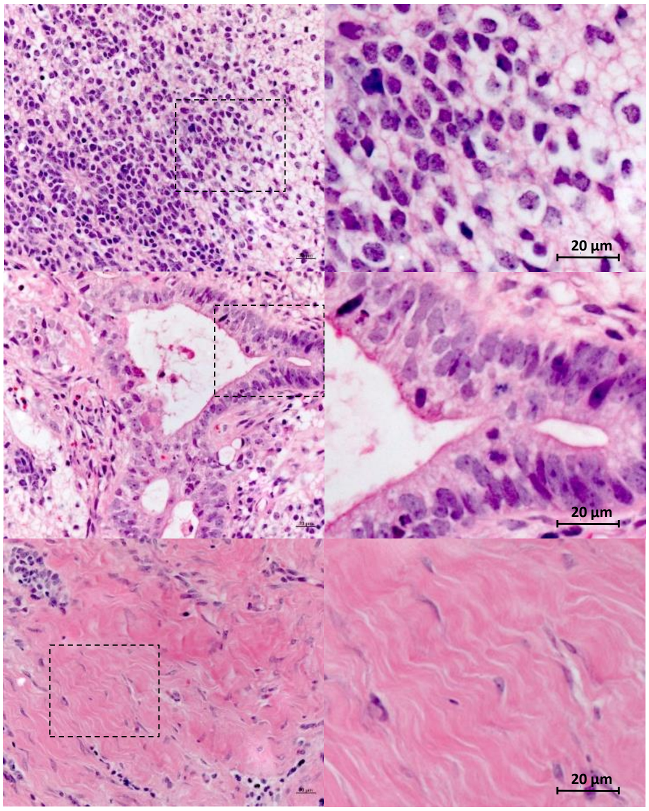

Ectoderm – nervous tissue

Endoderm – respiratory epithelium

Endo –Meso – fibrous connective tissue

Figure S3c

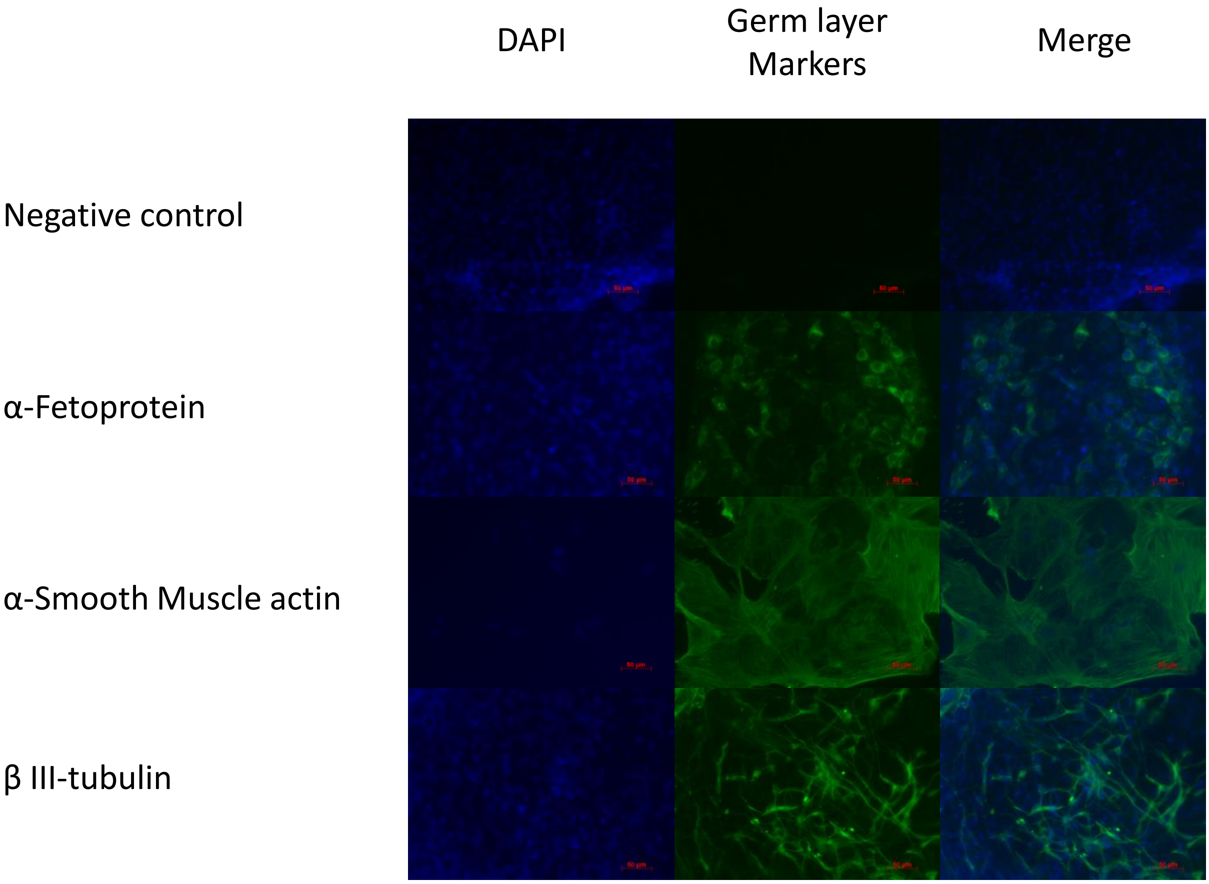

Figure S4: Embryoid bodies. Embryoid bodies were formed using the hanging drop method in 20% FBS for 10 days. Cells were fixed with paraformaldehyde, stained with indicated antibodies and counterstained with DAPI. Cells expressing α-feto-protein(endoderm), α -smooth muscle actin (mesoderm) and β-III-tubulin were detectable in 10-day EBs. Baboon iPSCs maintained in chemically-defined conditions are pluripotent

Supplementary Tables

Reagent	Provider	Catalog Number
IMDM with 1mM Glutamine	Biochrom	FG0465
RPMI 1640 with 1mM Glutamine	Gibco	61870
StemSpan SFEM	Stemcell Technologies	09650
Methyl- β -Cyclodextrin	Sigma	C4555
Trolox	Sigma	238813
Insulin	Sigma	I9218
Chemically defined Lipids 200X	Gibco	11905
Ethanolamine	Sigma	E0135
BSA	Gibco	From Kit A1000701
β-mercapto-ethanol 1000X	Gibco	21985
L-ascorbic acid	Sigma	A8960
Holo-Transferrin	R&D Systems/Biotechne	2914-HT
Optiferrin	FisherScience	NC9954311
FeIII-EDTA	Sigma	E6760
BMP4	R&D Systems/Biotechne	314-BP
VEGF165	Peprotech	100-20
Wnt3A	R&D Systems/Biotechne	5036-WN
Wnt5A	R&D Systems/Biotechne	645-WN
Activin A	Peprotech	120-14
GSK3β Inhibitor VIII	Calbiochem/EMD Millipore	361549
aFGF	Peprotech	100-17A
bFGF	Peprotech	100-18B
SCF	Peprotech	300-07
β-Estradiol	Sigma	E2758
TPO	Peprotech	300-18
IGF1	Alfa Aesar	BT-106
IGF2	Alfa Aesar	BT-107
SB431542	Tocris/Biotechne	1614
UM171	Stemcell Technologies	72912
IBMX	Sigma	I5879
PDGF AB	Peprotech	100-00AB
ANGPTL5	R&D Systems/Biotechne	6675-AN
CCL28	Peprotech	300-57
Heparin	Sigma	H3149
EPO	Amgen	NDC 55513-126-10
Dexamethasone	Sigma	D4902
RU486	Sigma	M8046
Hydrocortisone	Sigma	H0888
FLT3L	Peprotech	300-19
IL3	Peprotech	200-03
GM-CSF	Peprotech	300-03
G-CSF	Peprotech	300-23
TRA-1-81 (PE, mouse IgM)	eBioscience	12-8883-82
TRA-1-60 (PE, mouse IgM)	eBioscience	12-8863-82
SSEA-4 (FITC, mouse IgG3)	BD Pharmingen	560126
SSEA-3 (AF488, rat IgM)	eBioscience	53-8833-73
α-Fetoprotein (mouse IgG1)	R&D Systems	MAB1369
α-smooth Muscle Actin (mouse IgG2a)	R&D Systems	MAB1420
β-III Tubulin (mouse IgG2a)	R&D Systems	MAB1195
Goat anti-Mouse IgG (H+L), F(ab')2 Fragment (Alexa fluor 488)	Cell Signalling Technology	4408S

Table S1: Reagents

**Culture media and supplements**	**IMIT**<br>IMDM with 1mM Glutamine<br>Methyl-β-Cyclodextrin 0.1mg/mL<br>Trolox50µM<br>Insulin 10µg/mL<br>Optiferrin 50µg/mL<br>FeIII-EDTA 4µM<br>Chemically defined Lipids (1.5X)<br>Ethanolamine<br><br>**R6**<br>RPMI 1640<br>L-ascorbic acid 220 uM<br>Insulin 10ug/mL<br>Optiferrin 20 ug/mL<br>FeIII-EDTA 4µM<br>Chemically defined Lipids 0.5x	**S1**<br>BMP4 10ng/mL<br>VEGF 165 10ng/mL<br>Wnt3A/5A 5ng/mL each<br>Activin A 5ng/mL<br>Inhibitor VIII 2uM<br>bFGF 10ng/mL<br><br>**S2**<br>BMP4 20ng/ml<br>VEGF 165 30ng/mL<br>Wnt3A/5A 5ng/mL each<br>Activin A 5ng/mL<br>Inhibitor VIII 2uM<br>bFGF 10ng/mL<br>SCF 20ng/mL<br>β-Estradiol 0.4ng/mL<br><br>**S3**<br>BMP4 20ng/mL<br>VEGF 165 30ng/mL<br>bFGF 20ng/mL<br>SCF 30ng/mL<br>TPO 10ng/mL<br>IGF2 10ng/mL<br>β-Estradiol 0.4ng/mL<br>SB431542 3µM on day 3 only<br>IBMX 50 µM<br>UM171 30nM after day 6<br>Heparin 5µg/mL<br><br>**S4**<br>VEGF165 5ng/mL<br>bFGF 5ng/mL<br>SCF 15ng/mL<br>TPO 10ng/mL<br>IGF2 10ng/mL<br>IBMX 30 µM<br>PDGF AB 5ng/mL<br>ANGPTL5 5ng/mL<br>CCL28 5ng/mL<br>UM171 30nM<br>Heparin 5µg/mL	**SED**<br>SCF 100ng/mL<br>EPO 4U/mL<br>IBMX 50 µM<br>Dexamethasone 1 µM<br><br>**SER**<br>SCF 50ng/mL<br>EPO 4U/mL<br>RU486 1 µM<br><br>**SER2**<br>SCF 10ng/mL<br>EPO 4U/mL<br>RU486 1 µM<br><br>**R**<br>RU486 1 µM

Table S2: Cell Culture media and supplements
